# Niclosamide-Loaded Polyanhydride Nanoparticles to Combat Gemcitabine Resistance in Pancreatic Cancer

**DOI:** 10.1007/s40883-025-00394-0

**Published:** 2025-03-17

**Authors:** Brianna M. White, Venugopal Gunda, Susheel Kumar Nethi, Nagabhishek Sirpu Natesh, Adam S. Mullis, Mariaelena Roman Sotelo, Jeffrey North, Chris Destache, Balaji Narasimhan, Surinder K. Batra, Surya K. Mallapragada, Satyanarayana Rachagani

**Affiliations:** 1 Department of Chemical and Biological Engineering, Iowa State University, Ames, Iowa USA; 2 Department of Biochemistry and Molecular Biology, University of Nebraska Medical Center, Omaha, NE USA; 3 Department of Veterinary Medicine and Surgery, University of Missouri, Columbia, MO USA; 4 Roy Blunt Nextgen Precision Health, University of Missouri, Columbia, MO USA; 5 Department of Pharmacy Sciences, Creighton University, Omaha, NE USA; 6 Department of Pharmacy Practice, Creighton University, Omaha, NE USA; 7 Nanovaccine Institute, Iowa State University, Ames, Iowa USA; 8 Present Address: Department of Biomedical Engineering, Tufts University, Medford, MA USA

**Keywords:** Drug delivery, Pancreatic cancer, Nanoparticles, Biodegradable, Niclosamide

## Abstract

**Purpose:**

Pancreatic cancer (PC) is a highly lethal malignancy and lacks effective treatments. Current chemotherapies, including gemcitabine (Gem) in combination treatment regimens, produce dose-limiting toxicity, drug resistance, and ultimately limited improvement in the overall survival of PC patients. Niclosamide (Nic), a clinically safe FDA-approved anthelmintic drug has been shown to have anti-cancer properties; however, its limited bioavailability makes Nic largely ineffective as a therapeutic agent. To address this challenge, we have developed a novel combination therapy of Gem with the repurposed drug, Nic, loaded in biodegradable polyanhydride nanoparticles (NicNp), as an effective treatment option for PC.

**Methods:**

We synthesized and characterized NicNp in vitro and evaluated their biodistribution and efficacy in xenograft and syngeneic pancreatic tumor models in mice.

**Results:**

The biodistribution study indicated that NicNp accumulated in high concentrations in the pancreatic tumors of the mice with C_max_ of 138 ± 74.1 µg Nic/g tissue. NicNp treatment, in combination with Gem, worked synergistically to reduce the dose of gemcitabine required to kill pancreatic cancer cells in vitro*,* two-fold*.* Additionally, the pancreatic tumor burden in the mouse models was significantly reduced, while survival was significantly increased when mice bearing pancreatic tumors were treated with the combination of NicNp and Gem.

**Conclusions:**

This study demonstrates the potential for effective repurposing Nic via nanoformulations in combination with Gem to improve PC treatment efficacy.

**Lay summary:**

Pancreatic cancer (PC) ranks among the most lethal types of cancer, with largely ineffective current treatments and toxic side effects in patients. Niclosamide is an FDA-approved anti-parasitic drug with minimal side effects, that has shown some anti-cancer properties. However, it is not effectively absorbed in the body. We produced polymer nanoparticles to deliver niclosamide effectively to treat pancreatic tumors in mice in combination with the chemotherapeutic gemcitabine. This combination treatment led to PC tumor reduction and increased the survival, demonstrating that niclosamide encapsulated in nanoparticles in combination with gemcitabine has the potential to be a more effective treatment for PC.

**Graphical Abstract:**

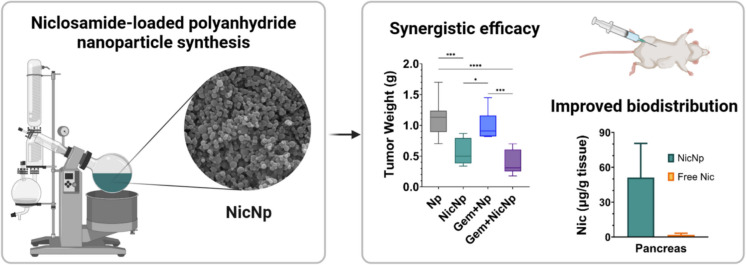

**Supplementary Information:**

The online version contains supplementary material available at 10.1007/s40883-025-00394-0.

## Introduction

In the past 5 decades, the survival rate of pancreatic cancer (PC), ranked as the 4th major contributor to cancer-related fatalities, has seen only a modest increase from 3 to 12% [1]. Pancreatic cancer is aggressive, and clinical outcomes have lagged due to the lack of early detection and due to largely ineffective treatments [2, 3]. Currently, chemotherapy is an essential treatment for PC patients, and the most common first-line chemotherapy options for PC patients are gemcitabine (Gem), Gem/nano-albumin-bound paclitaxel (nab-paclitaxel), or FOLFIRINOX [4, 5]. Although FOLFIRINOX demonstrated enhanced survival rates in advanced-stage pancreatic cancer patients compared to Gem and Gem/nab-paclitaxel, its significant toxicity renders it intolerable for many PC patients [6]. Gemcitabine is an antimetabolite drug that targets cancer cells by inhibiting nucleotide metabolism, but its efficacy is negated by multiple complex mechanisms that promote tumor progression [7–9]. Patients treated with Gem often become resistant to its therapeutic effect, which leads to disease progression and failed treatment. Novel therapeutic combinations that target multiple oncogenic mechanisms are needed to reduce chemotherapy resistance, such as that seen with Gem treatment, and overall survival of PC patients [10].

Clinical trials and pre-clinical studies with combination treatments using two or more drugs often result in dose-limiting systemic toxicity and disease recurrence in therapy-resistant PC patients [11–13]. This emphasizes the need to focus on drug combinations with low toxicity and the ability to suppress chemoresistance [14]. Various drugs initially developed for treating non-oncological diseases possess anti-cancer properties and could be repurposed as cancer treatments [15]. With established safety profiles, these drugs offer new treatment regimens for cancer, promising speedier development, enhanced efficacy, and reduced financial burden compared to conventional drug discovery avenues [15].

Niclosamide (Nic) is an anthelminthic salicylanilide drug approved by the Food and Drug Administration (FDA) in 1982 and has garnered attention for repurposing. Anti-neoplastic evaluations of Nic in various cancer models reveal its ability to tackle diverse oncogenic pathways [16–19]. The primary target of Nic is mitochondrial uncoupling, as Nic disrupts oxidative phosphorylation, metabolism, and ATP in targeted organisms [20] and in cancer cells [21]. Our group reported that Nic inhibits PC cell growth by inhibiting the non-canonical pathway of Shh/Gli3 and causing mitochondrial damage [18]. When given orally at a high dosage of 2 g daily, Nic has not shown any significant toxic effects for patients with cestode infections [22]. The favorable safety profile of Nic, coupled with its ability to inhibit several vital cellular signaling mechanisms, makes it a promising PC therapeutic. However, poor intestinal absorption and low bioavailability due to Nic’s hydrophobicity have confined its evaluation to colorectal cancer of the luminal cavity. Hence, there is a pressing need for Nic formulations that could offer enhanced bioavailability to broaden its potential application in PC treatment [23, 24].

Nanoparticle mediated drug delivery strategies assist drugs with poor bioavailability to overcome physiological barriers and improve biodistribution and bioavailability of hydrophobic drugs [25]. Nab-paclitaxel is an FDA-approved nanoformulation that has improved delivery of the hydrophobic drug paclitaxel for PC treatment [26, 27]. While nanoformulations of Nic have been recently explored for lung, breast, prostate, and colon cancer [24, 28, 29], studies evaluating Nic nanoformulations for PC treatment are currently lacking. Polyanhydride nanoparticles are a promising family of drug delivery carriers that are biodegradable and biocompatible and can provide a tunable release (via alteration of copolymer composition) of encapsulated small molecule payloads [30, 31]. These nanocarriers have demonstrated enhanced delivery and improved potency of the hydrophobic drugs ivermectin and rifampicin [32, 33].

Based on these previous successes of polyanhydride nanoparticle delivery of highly hydrophobic drugs, we synthesized polyanhydride nanoparticles loaded with Nic (NicNp) and investigated their potential for treating PC in combination with Gem. This study demonstrated that our NicNp formulation significantly improved bioavailability and enhanced the efficacy of Nic treatment for PC compared to free Nic. Most significantly, we showed that NicNp sensitized PC in orthotopic tumor-bearing mice to Gem synergistically, and significantly improved survival and tumor burden.

## Materials and Methods

### Polyanhydride Synthesis and Characterization

The anhydride monomers for this study, 1′6-bis(carboxyphenoxy) hexane (CPH) and sebacic acid (SA), were chosen based on previous release studies using 20:80 CPH:SA with hydrophobic drugs, which showed release over the course of 1 week [30, 33]. Sebacic acid (Fisher Scientific, Waltham, USA) and CPH diacid were synthesized as previously described, [34]. As in previous studies, poly (CPH-co-SA) random copolymers with a molar ratio of 20:80 CPH:SA were synthesized via melt condensation [30]. First, CPH diacid and sebacic acid were acetylated in excess acetic anhydride at 125 °C for 30 min. Excess acetic anhydride was removed by rotary evaporation. Then, the acetylated monomers were polymerized in an oil bath at 180 °C under vacuum (< 0.3 torr) for 30 min. The copolymer was then dissolved in methylene chloride and precipitated in chilled hexanes. The hexanes were removed via vacuum filtration, and the copolymer was dried overnight under vacuum. The copolymer composition and number average molecular weight (Mn) were characterized via ^1^H NMR analysis using a Varian MR-400 (Varian Inc., Palo Alto, USA). End-group analysis of the integrated ^1^H NMR spectra was used to calculate the number-average molecular weight, Mn.

### Nanoparticle Synthesis and Characterization

Niclosamide-loaded polyanhydride nanoparticles were synthesized via flash nanoprecipitation, applying a method previously described [30], to encapsulate the Nic in the polymer nanoparticle, as opposed to chemically attaching it. The nanoprecipitation was performed in a cold room with pre-chilled pentane (−20 °C) as antisolvent and 4:1 methylene chloride to methanol as solvent. The copolymer concentration in solvent was 20 mg/mL, and the solvent-to-antisolvent ratio was 1:200. The initial mass ratio of Nic to copolymer was varied to determine the loading capacity of the nanoparticles. The NicNp were dried under vacuum following filtration using an aspirator vacuum and stored with desiccant in a −20 °C freezer. An FEI Quanta 250 FEG scanning electron microscope (SEM) was used to image the NicNp, and the SEM images of NicNp were analyzed for size using Fiji image analysis software. The Zeta potential of the NicNp in phosphate-buffered saline (PBS) at pH 7.4 and 37 °C using a Zetasizer Nano ZS90 (Malvern Instruments Ltd., Worchester, UK).

### Encapsulation Efficiency and Release Studies

HPLC–UV (1200 series system, Agilent Technologies, Santa Clara, USA) was used to quantify the encapsulation efficiency, loading, and release kinetics of Nic from NicNp. The encapsulation efficiency and loading of Nic in NicNp were determined using two methods. First, the NicNp were dissolved in chloroform and separated via NP-HPLC on a Zorbax Rx-SIL 5 mm, 4.6 × 150 mm column using a gradient from 0.1: 99.9 methanol to chloroform to 99.9: 0.1 over 22 min at a flow rate of 1 mL/min. Elution of Nic was measured at 335 nm at 2.2 min, while polymer elution was monitored at 260 nm. Second, a base extraction was conducted to degrade the polymer quickly by adding 40 mM sodium hydroxide and incubating at 37 °C on a shaker set to 100 RPM until the nanoparticles completely eroded. The Nic from the samples was measured with RP-HPLC using a Zorbax Eclipse XDB-C8 5 um, 4.6 × 150 mm column using a gradient from 25:75 nanopure water 0.1% trifluoroacetic acid to methanol 0.1% trifluoroacetic acid to 0.1:99.9 over 12 min at a flow rate of 1 mL/min. For these samples, Nic elution at 13.9 min was measured at 335 nm, while the polymer was detected at 260 nm. Drug loading was calculated by dividing the mass of Nic released by the initial mass of nanoparticles in each sample. Encapsulation efficiency was calculated by dividing the initial (at synthesis) mass ratio of Nic to polymer by the final (released) mass ratio.

Release studies were conducted by weighing approximately 3 mg of NicNp into microcentrifuge tubes. Phosphate-buffered saline (pH 7.4) was added to each sample and briefly sonicated to form a suspension. The samples were covered in an incubator at 37 °C with a shaker set to 100 rpm. At predetermined time points, the samples were removed from the incubator and centrifuged for 5 min at 15,000 RCF. The supernatant was collected for analysis, and methanol was added as a wash step to dissolve any Nic that was not dissolved in the PBS due to the hydrophobicity of Nic. Samples were centrifuged again, and the supernatant was collected for analysis. PBS was added to the samples, which were then briefly sonicated to form a suspension and returned to the incubator until the next time point. Supernatants of PBS and methanol were measured on RP-HPLC using the same method mentioned previously for base extractions. The total area under the peak for both PBS and methanol wash was added at each time point to determine the total amount of Nic released at each time point.

### Cytotoxicity Assays

Cytotoxicity of NicNp and Nic treatment in PC cell lines was assessed by an MTT assay. Briefly, 1 × 10^4^ -1 cells or KCT-3248 cells were seeded in 96-well plates and incubated overnight at 37 °C under 5% carbon dioxide. Then, the cells were incubated with media containing NicNp or free Nic at doses of 0 to 10 µM Nic for 72 h. The treatments were then removed from the well plates, and fresh media containing 0.5 mg/mL MTT (3-(4,5- dimethylthiazol-2-yl)−2,5-diphenyltetrazolium bromide) (Promega, Madison, USA) was added to the plates. The cells were incubated for an additional 3 h before removing the MTT solution and adding the stop solution. The absorbance was measured at 570 nm, and the untreated control absorbance was used to calculate the relative survival of treated cells.

### Niclosamide Biodistribution in Orthotopic Tumor Models of Pancreatic Cancer

The tumors were innoculated by orthotopically implanting 0.25 million AsPC-1 or KCT-3248 cells into the pancreas of athymic nude mice and C57BL6 mice following the experimental animal protocol approved by the University of Nebraska Medical Center’s Institutional Animal Care and Use Committee (protocol code 20–091-11-FC). The AsPC-1 cell line is derived from ascitic fluid of a 62 year-old female with PC and are naturally resistant to Gem treatment [35]. The KCT-3248 cell line was derived from the pancreatic tumors of KPC mice and is also resistant to Gem treatment. Studies with AsPC-1 cells were conducted in immunocompromised athymic nude mice, while immunocompetent C57BL6 mice were used with the KCT-3248 cell line. Mice bearing fully developed AsPC-1 and KCT-3248 derived tumors (10 days post-implantation) were injected intraperitoneally (IP) with 300 µL NicNp resuspended in PBS containing 1.8 mg Nic. Additionally, a group of mice was injected with 1.8 mg free Nic in PBS. Mice were then euthanized at time points ranging from 5 min up to 72 h post-treatment using CO_2_ asphyxiation followed by cervical dislocation. Blood, primary tumor, and distant organs (liver, kidney, heart, and brain) were collected at the time of euthanasia and flash-frozen in liquid nitrogen. Whole blood was collected in a K_2_EDTA collection tube (BD Microtainer MAP, Franklin Lakes, USA) and centrifuged at 394 × g for 20 min to harvest the plasma; the plasma was then frozen until analyzed. GraphPad Prism^▯^ was used to determine the pharmacokinetic (PK) parameters from plasma and tissues using noncompartmental modeling of the concentration–time data. To quantify Nic amounts, 500 µL of the processed sample was injected into an LC–MS/MS apparatus (5500 QTrap; AB Sciex, Framingham, USA) operated in electrospray ionization (ESI) negative mode controlled by Analyst 1.6.1 software. The chromatographic separation was performed on a Phenomenex Kinetex column (50 × 3 mm, 5 mm) with a mobile phase composed of 10 mM ammonium formate in water and acetonitrile 30:70 (vol/vol) at a flow rate of 0.3 mL/min. The mass transitions 325.0—288.8 and 325.0 – 170.8 were used for Nic and 306.7 – 160.8 for internal standard (warfarin, 0.5 µg/mL), respectively. The calibration range was 23.43—3000 ng/mL. The chromatographic run time for each sample was 3 min with retention times of 0.78 min (warfarin) and 1.14 min (Nic). Inter-day and intra-day variability was < 10%.

Safety analysis was performed in the mice taken at the 24 h timepoint of the biodistribution study. Plasma (50 µL) separated from blood samples of the euthanized mice was utilized for estimating enzyme activities and safety parameters including blood urea nitrogen (BUN), alanine transaminase (ALT), total bilirubin (TBIL), glucose, total protein (TP), albumin, globulin, alkaline phosphatase (ALP), aspartate transferase (AST), CO_2_, potassium and creatinine levels using Abaxis VetScan VS2 chemical assay discs (Zoetis, Parsippany, USA).

### Drug Synergism and Combination Index Calculation

The synergistic effect of Gem and NicNp combinations on PC cells was determined by combination-index methods, derived from the median effect principle of Chou and co-workers (*59*), using the Compusyn software to determine the combination-index (CI) between the two drugs. The AsPC-1 cells were seeded and incubated overnight as described for cytotoxicity assays. Subsequently, the cells were incubated with Gem or NicNp in a concentration ranging from 0.25–6.67 µM or a combination of Gem and NicNp at 1:1, 1:2, and 2:1 ratios. Similarly, the combination index of Gem with Nic in different forms (either dissolved in PBS or DMSO or as Np) was also used to identify the best combination. After 72 h of incubating cells with the treatments, the media was replaced with fresh media containing MTT (3-(4,5- dimethylthiazol-2-yl)−2,5-diphenyltetrazolium bromide) (0.5 mg/mL) for 3 h. Finally, the MTT solution was removed, the stop solution was added to the cells, and the absorbance of the plates was recorded at 570 nm. The growth inhibition caused by the treatments was calculated with respect to the untreated controls. Triplicate experiments were performed and averaged. The growth inhibitory values obtained from the MTT assay were used to calculate the combination index (CI) values using Compusyn software, and the CI plots of these combinations were utilized to calculate CI values. A CI value of 1 is indicative of an additive effect between Gem and NicNp, whereas a CI < 1 and CI > 1 indicate synergism and antagonism, respectively.

### Dose Escalation and Efficacy Studies in Pancreatic Tumor Mouse Model

Therapeutic efficacy studies were performed in mice with tumors derived from orthotopic implantation of AsPC-1 and KCT-3248 cells, as described in the biodistribution studies. Free Nic, Np, or NicNp (containing 1.8 mg Nic) were resuspended in sterile saline and sonicated as described in the cell survival assays. The mice were injected IP once weekly for 3 weeks with their respective treatments. In a separate dose escalation study, orthotopic tumor-bearing mice were injected IP with 200 mL, 300 mL, or 500 mL of NicNp containing 1.2 mg, 1.8 mg, and 3.0 mg Nic, respectively, once weekly for four weeks. We also performed therapeutic efficacy of NicNp in combination with Gem therapy in mice bearing palpable orthotopic tumors derived from AsPC-1 and KCT-3248 cells. The mice were divided into four groups (*n* = 8). The control group was injected IP with 300 µL Np resuspended in PBS, Gem + Np group received 300 µL Np and 50 µL Gem (25 mg/kg/body weight), NicNp group was injected IP with 300 µL NicNp containing 1.8 mg Nic and Gem + NicNp group were injected with 300 µL NicNp and 50 µL Gem (25 mg/kg/body weight), resuspended in PBS once weekly for 4 weeks. Mice were provided with a normal diet and water ad libitum and monitored for tumor growth as well as active feeding and behavior. Mice either bearing tumors beyond the permissible size or showing distress and signs of discomfort were considered non-alive and euthanized for tumor necropsies. Mice were treated for four weeks and euthanized by CO_2_ asphyxiation followed by cervical dislocation for collecting primary tumors, organs, and blood for further analyses.

Tissue Sects. (4 µm) obtained from the pancreatic tumors were embedded in paraffin, fixed on slides with paraformaldehyde, and stained with hematoxylin and eosin. In addition, immunohistochemistry was performed as described previously [36, 37] using anti-cleaved caspase-3 (9694S, Cell Signaling Technologies, Danvers, USA) and Anti-Ki67 antibody (ab15580, Abcam, Cambridge, UK) antibodies in mouse xenograft tumor sections from four treatment groups. Finally, the terminal deoxynucleotidyl transferase dUTP nick end labeling (TUNEL) assay was performed on tumor tissue sections. A commercially available kit (ab206386, Abcam, Cambridge, UK) was used to detect apoptotic cells in the mouse pancreatic tumor tissue to determine efficacy of therapy. Briefly, 5 µm thick tissue sections were deparaffinized in xylene and dehydrated with graded alcohol, followed by proteinase-K treatment and 3% H_2_O_2_ treatment to inactivate endogenous peroxidases in the cells. Biotin-labeled deoxynucleotide incorporation in apoptotic cells catalyzed by the terminal deoxynucleotidyl transferase (TdT) was detected by incubating with streptavidin–horseradish peroxidase (HRP) conjugate. The signals were detected by 3,3′-diaminobenzidine (DAB) substrate, and sections were further counterstained with methyl green. Positive and negative control tissue treated with DNase I and water instead of TdT were used for comparison.

### Statistical Analyses

Biological triplicate data from in vitro assays and in vivo studies were analyzed for mean ± standard error of the mean (SEM) with Students-t-tests comparing two data sets and one-way analysis of variance (ANOVA) for multiple comparisons. These data analyses, including normalization and significance testing, were performed using either Excel (Microsoft) or Prism 9 (GraphPad Software Inc.), considering p values < *0.05, **0.01, and ***0.001 as significant.

## Results

### Nanoparticle Characterization and In Vitro Toxicity

The characterization of Nic-loaded polyanhydride nanoparticles (NicNp) involved polymer molecular weight determination followed by optimization of the NicNp formulation. End group analysis of ^1^H NMR spectra of the polyanhydride random copolymers poly (CPH-co-SA) indicated the number-average molecular weight, M_n_, to be approximately 17 kDa and confirmed the molar ratio of 20:80 CPH:SA in the copolymers (Fig. S1). An increasing mass of Nic, ranging from 10–40 mg Nic and 100 mg polymer was used to synthesize NicNp to determine the maximum effective loading. Loading of Nic by weight ranged from 0.5% to a maximum of 12% Nic. NicNp with a higher initial mass of Nic (30–40 mg) resulted in the formation of Nic crystals outside of the Np, so the optimal initial mass ratio of Nic to polymer was determined to be 1:5. The encapsulation efficiency averaged from both normal phase and reverse phase HPLC–UV measurements was 60% ± 10%.

**Fig. 1:**
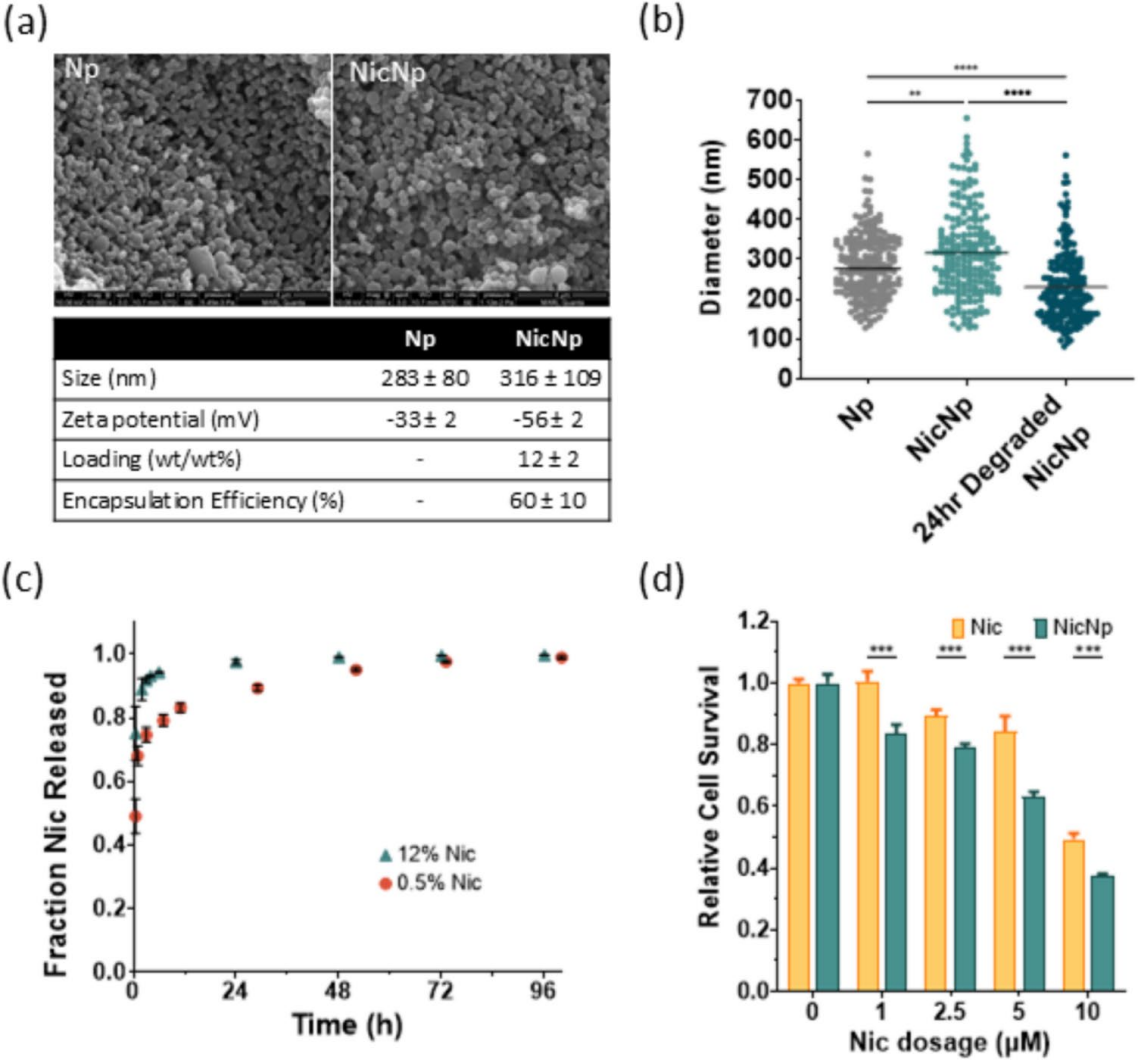
In vitro characterization of NicNp. (**a)** SEM micrograph of Np and NicNp with a table including the average ± SD diameter, zeta potential, Nic loading, and encapsulation efficiency of Np and NicNp (**b**) distribution of the diameters of NicNp and NicNp post-incubation in PBS for 24 h with the line at the mean diameter (*n* = 200). (**c**) cumulative Nic released from NicNp in vitro in PBS (pH 7.4) with different Nic loading percentages, (**d**) relative cell survival of AsPC-1 cells treated for 72 h with Nic and NicNp. Error bars represent mean ± SD of 3 or more replicates. **, ***, **** represent *p* < 0.01, *p* < 0.001 and 0.0001 respectively

The release kinetics of Nic from NicNp with two different loadings (%wt/wt) of Nic, 12% and 0.5%, were measured. Release studies of 12% NicNp in PBS showed a burst release (89%) in the first two hours, followed by the release of the remaining Nic over 48 h (Fig. 1c). In the 0.5% Nic loading case, NicNp showed a slower release in the first 2 h of ~ 70%, while the remaining Nic was released over 4 days (Fig. 1c). Thus, 12% NicNp loading was chosen for the subsequent experiments to maximize mass of delivered Nic, while minimizing total nanoparticle mass (therefore injection volume) in subsequent in vivo studies. Scanning electron microscopy (SEM) image analysis of Np and NicNp showed that the particles are spherical and polydisperse, with sizes ranging from 283 nm ± 80 nm and 316 nm ± 109 nm, respectively (Fig. 1a & b). Additionally, NicNp degraded to 70% of their original size after the first 24 h incubated in PBS at 37 °C (Fig. 1b). This degradation is expected because anhydride bonds in the polymer chain are broken by hydrolysis. The zeta potential of NicNp in PBS (pH 7.4) was −56 mV ± 2 mV compared to empty Np at −33 mV ± 2 mV.

Following the optimization and characterization of the NicNp formulation, cell viability analysis was conducted on AsPC-1 cells treated with free Nic and NicNp at different concentrations using the MTT assay. The survival rate of AsPC-1 cells exhibited a dose-dependent response to both free Nic and NicNp. However, NicNp demonstrated a significant reduction in cell survival compared to free Nic across all tested dosages ranging from 1 µM to 10 µM. (Fig. 1d).

### Tissue Distribution and Toxicity of Nic-Loaded Nanoformulations In Vivo

**Fig. 2:**
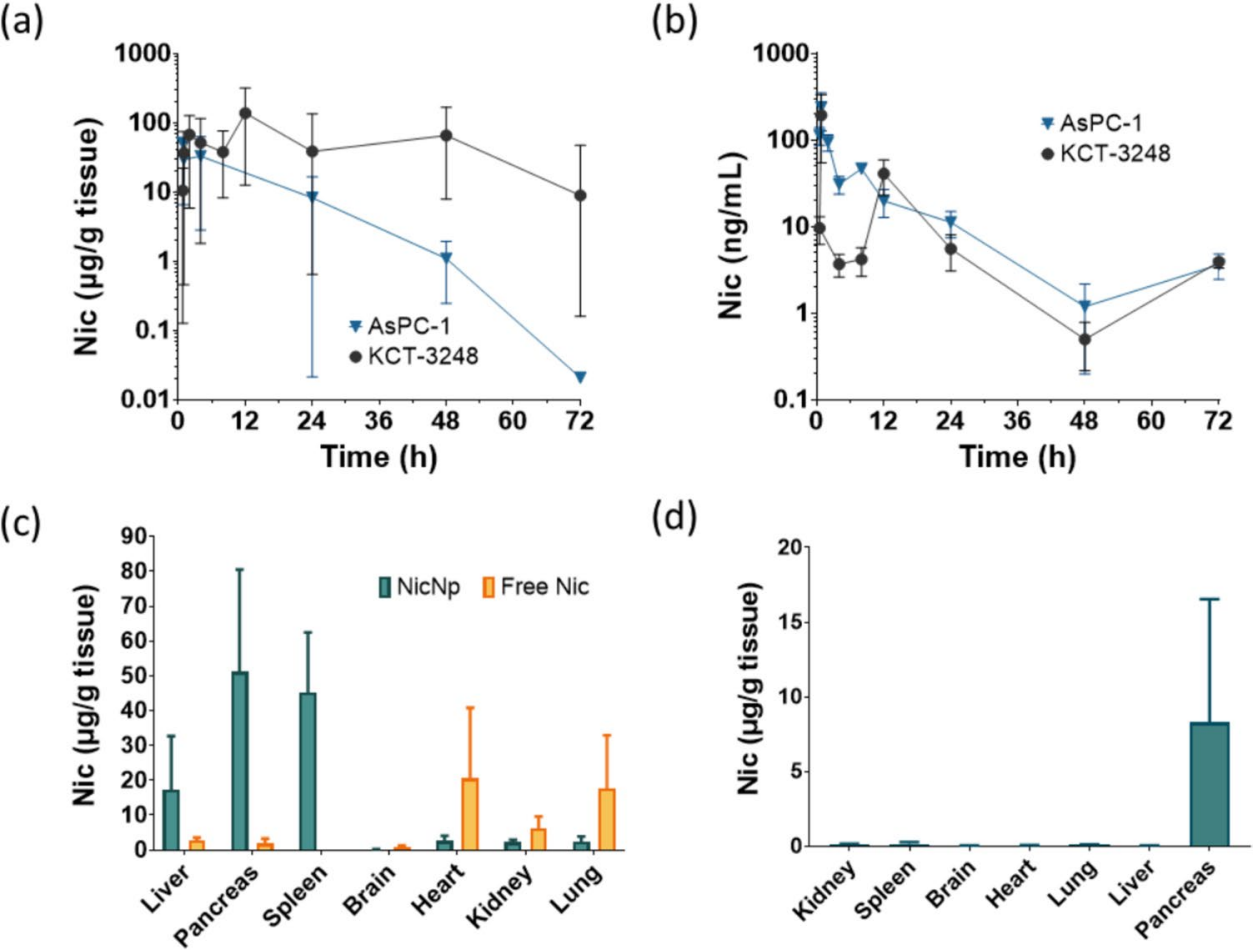
Biodistribution and serological effects of Nic in NicNp-treated mice**.** Niclosamide concentration in (**a**) pancreatic tumor and (**b**) plasma of NicNp-treated mice bearing AsPC-1 and KCT-3248 tumors, (**c**) Nic concentration in organs from free Nic and NicNp-treated KCT-3248 tumor-bearing mice, (**d**) Nic concentration in organs from NicNp-treated AsPC-1 tumor bearing mice. Bar graphs represent mean ± SEM data derived from 3–10 biological replicates, * represents *p* < 0.05

Following synthesis, characterization, and in vitro cytotoxicity assays, biodistribution studies were performed in mice bearing xenograft tumors derived from orthotopic implantation of KCT-3248 and AsPC-1. NicNp was administered IP and at specific time points, the mice were euthanized. Plasma and organs were collected from the mice and the concentration of Nic was measured. The concentration of Nic in pancreatic tumors was detected within 5 min after administration of NicNp. In KCT-3248 tumor-bearing mice, the T_max_ in the pancreas was 12 h with a C_max_ of 138 ± 74.1 µg/g tissue (Fig. 2a). In AsPC-1 tumor-bearing mice, the T_max_ in the pancreas was 5 min with a C_max_ of 51.4 ± 23.0 µg/g tissue (Fig. 2b). The total area under the concentration–time curve (AUC) is used to measure the exposure of a given tissue or plasma to Nic. The average pancreas/tumor AUC_0–72h_ was 3,920 ± 1,820 µg/g in KCT-3248 mice and 640 ± 666 µg/g in AsPC-1 mice, respectively (Fig. 2a).

The concentration of Nic in pancreatic tumors was significantly higher than plasma concentrations at all time points in both KCT-3248 and AsPC-1 tumor-bearing mice (Fig. 2a and b). The time to maximum Nic (T_max_) concentration in plasma after IP injection of NicNp in both KCT- 3248 and AsPC-1 tumor-bearing mice was 5 min (Fig. 2b). The maximum concentrations of Nic (C_max_) in plasma at that time were 194 ± 140 ng/mL and 239 ± 111 ng/mL in KCT-3248 and AsPC-1, respectively (Fig. 2b). The plasma AUC_0–72h_ averaged 858 ± 456 ng/mL for KCT-3248 and 1100 ± 194 ng/mL for AsPC-1 tumor-bearing mice (Fig. 2b). The concentration of Nic in vascular organs and in the brains from mice bearing KCT-3248 tumor were compared at 24 h post-treatment with NicNp and free Nic. The concentration of Nic measured in the spleens from NicNp group at 24 h post-injection was significantly higher than that of Nic measured in the spleens of KCT- 3248 tumor-bearing mice injected with free Nic (Fig. 2c). The Nic concentration was also higher in the liver and pancreas of NicNp-treated mice compared to free Nic-treated mice (Fig. 2c). Free Nic concentration was higher in the non-intraperitoneal organs (brain, lungs, kidneys, and heart compared to Nic from NicNp (Fig. 2c). Mice with AsPC-1 tumors injected IP with NicNp did not show a significant difference in Nic concentration between organs (Fig. 2d).

The plasma safety parameters from mice bearing KCT-3248 cell-derived xenograft PC tumors treated with NicNp and free Nic for 24 h indicate that Nic treatment demonstrated deviations from the Np treated group in BUN, glucose and potassium levels, however; these levels were still within what would be considered a normal range based on work from Otto et al. and Silva-Santana et al. (Fig. S2) [38–40].

### Niclosamide Nanoparticles are More Effective than Free Nic in Treating PC Tumor-Bearing Mice

**Fig. 3:**
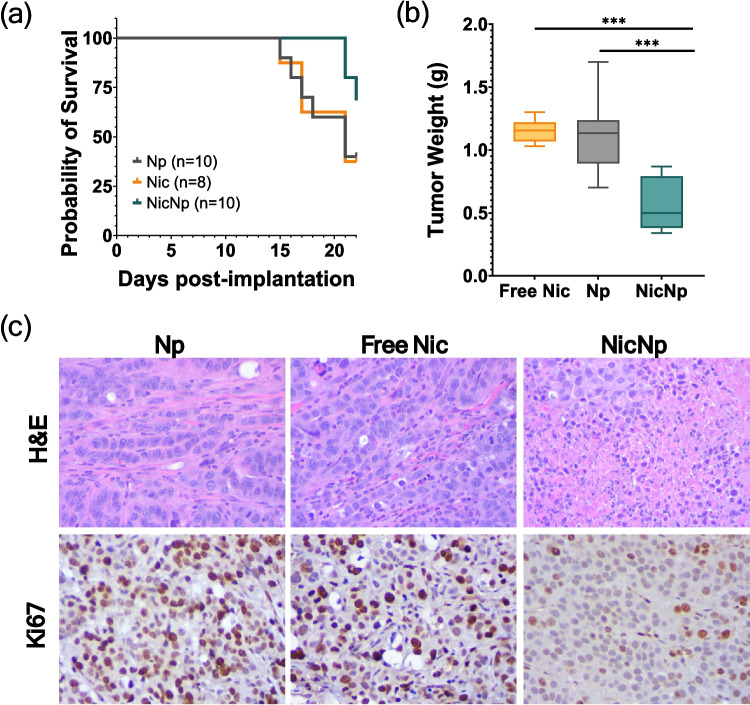
Nanoformulated Nic extended survival and reduced tumor weight in KCT-3248 tumor-bearing mice. (**a**) Probability of survival in KCT-3248 tumor-bearing mice, (**b**) reduction in tumor weights in mice treated with NicNp compared to Np and free Nic, *** represents *p* < 0.001 from One-way ANOVA (*n* = 8), (**c**) representative histological images of reduced tumor cell density (blue) by H&E staining and reduced proliferation markers by Ki67 staining (brown) in Np, free Nic, and NicNp treated mice tumors. Histological images were captured at 20 × magnification

Therapeutic efficacy of NicNp was evaluated using pancreatic tumors derived from orthotopic implantation of AsPC-1 cells in athymic nude mice and KCT-3248 cells in syngeneic immunocompetent C57BL6 mice. To identify the minimum effective dosage of NicNp without toxicity, survival and tumor burden were analyzed in KCT-3248 tumor-bearing mice injected with increasing doses of NicNp. The study showed improved survival and reduced tumor burden in mice after a dosage of 15 mg NicNp, which contains 1.8 mg Nic, was injected IP (Fig. S3). Following the dose escalation study, KCT-3248 tumor-bearing mice were treated weekly with free Nic, Np, and NicNp. A dosage of 1.8 mg free Nic and 15 mg NicNp (equivalent to 1.8 mg Nic) was selected based on the dose escalation study results. The NicNp-treated group showed significantly increased survival compared to free Nic as well as Np (Fig. 3a). Additionally, the tumor weights of NicNp-treated mice were significantly reduced compared to both free Nic and Np (Fig. 3b). Histological staining of tumor sections with hematoxylin and eosin (H&E) showed reduced mitotic index, hypo-nuclear and reduced cellular density in the NicNp treated tumors compared to the free Nic treated tumors (Fig. 3c). Ki67 is a proliferative marker and was used to assess the ability of NicNp treatment to reduce cancer cell proliferation [41, 42]. Ki67 showed reduced intensity in NicNp-treated tumors compared to free Nic and empty Np-treated tumors (Fig. 3c). There were no significant differences in survival, tumor weights or histological staining in the free Nic and Np treated mice groups. These results, taken together, demonstrated that NicNp treatment can effectively increase survival and decrease tumor burden compared to free Nic.

### Nanoformulated Niclosamide Sensitizes Pancreatic Tumors to Gemcitabine

**Fig. 4:**
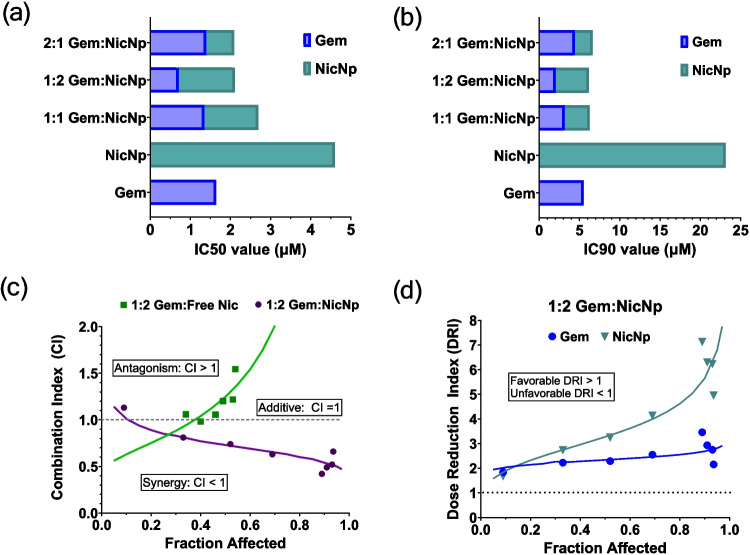
Combination treatment of AsPC-1 cells with Gem and NicNp show synergism. IC50 values (**a**) and IC90 values (**b**) of Gem + NicNp treatment combinations in AsPC-1 cells, (**c**) combination index of 1:2 Gem:Free Nic and Gem:NicNp in AsPC-1 cells, (**d**) dose reduction index of 1:2 Gem:NicNp in AsPC-1 cells

After confirming that NicNp is more effective in treating PC than free Nic, drug synergism of treatment combinations of Gem and NicNp were evaluated. The AsPC-1 cells were treated with constant ratios of 2:1, 1:1, and 1:2 Gem to Nic contained in NicNp to study the potential of NicNp to sensitize PC cells to Gem. The IC50 and IC90 values of the combinations were determined using the Compusyn software (Fig. 4a and b). The IC50 of NicNp alone was 4.6 µM while the Gem IC50 was 1.6 µM (Fig. 4a). At each ratio tested, the amount of Gem required to reach the IC50 was significantly reduced, compared to Gem alone (Fig. 4a). The most effective ratio to achieve the IC50 was 1:2 Gem:NicNp, which reduced the Gem dosage required by more than half compared to Gem only (Fig. 4a). Accordingly, the amount of NicNp required to reach IC50 at the 1:2 Gem:NicNp ratio was reduced from 4.6 µM to 1.4 µM (Fig. 4a). The same trend was shown at the IC90 for each combination ratio but with an even larger dose reduction (Fig. 4b). Again, the 1:2 Gem:NicNp combination had the largest decrease in Gem dosage required to reach the IC90 going from 5.5 µM Gem alone to 2.0 µM in the combination (Fig. 4b). The NicNp alone required to reach IC90 was dramatically reduced from 23.1 µM to 4.1 µM when used in combination at 1:2 Gem:NicNp (Fig. 4b).

To confirm that NicNp is more effective than free Nic when used in combination with Gem, AsPC-1 cells were treated with a combination of Gem with free Nic and Gem with NicNp at a ratio of 1:2 and comprising 1.5, 2.25, 3, 4.5, 6, 7.5, and 10 µM total concentration. The combination index (CI) is a quantitative measure of the synergism of 2 or more drugs used in combination at constant ratios. Synergism is defined as CI below 1, while CI values above 1 are considered antagonistic. The fraction affected (Fa) in these experiments is the fraction of cells killed. The combination treatment of 1:2 Gem with free Nic was antagonistic at almost all concentrations tested. In contrast, the combination treatment of 1:2 Gem with NicNp showed synergism at all but one concentration tested (Fig. 4c). The 2:1 and 1:1 ratios of Gem to NicNp in AsPC-1 cells only showed synergism at the higher total concentrations (Fig. S4). Dose reduction index (DRI) is the fold reduction of the dose of a drug used in a combination compared to its dose used alone required to reach a specific Fa. The DRI for Gem and NicNp were favorable at all the concentrations tested in the 1:2 Gem:NicNp combination (Fig. 4d). The dose reduction for Gem was approximately twofold across the entire range of Fa (Fig. 4d). The DRI for NicNp increased significantly as Fa increased going from 0.6 at 0.01 Fa to 7 at 0.94 Fa (Fig. 4d).

**Fig. 5:**
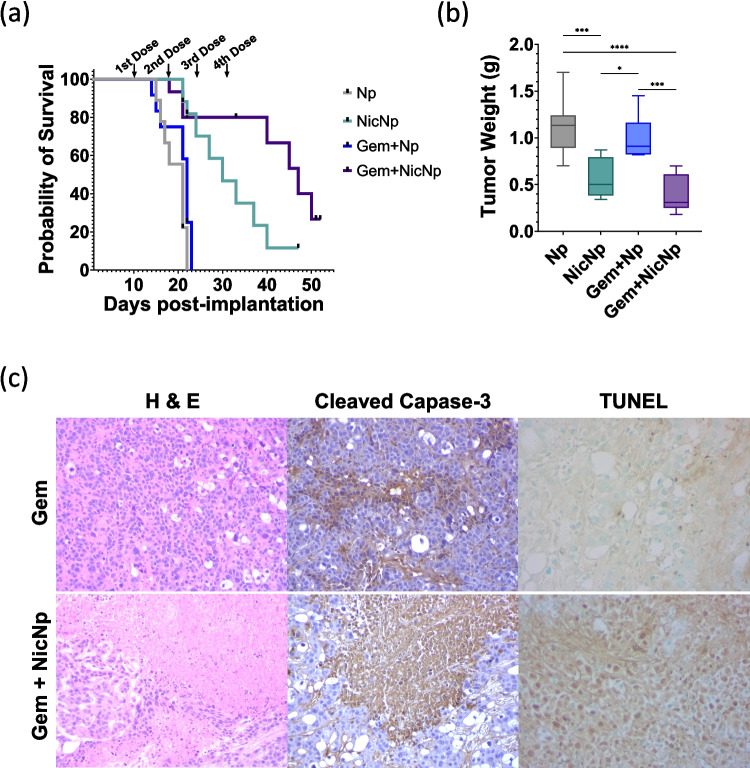
Combination treatment of NicNp + Gem significantly improves survival and reduces tumor weight in KCT-3248 tumor-bearing mice. (**a**) Survival of KCT-3248 tumor-bearing mice (**b**) reduced tumor weight in NicNp and Gem + NicNp treated KCT-3248 tumor-bearing mice at the time of necropsy (*n* = 7 for Gem + Np and *n* = 8 for all other groups), (**c**) representative histological micrographs showing alterations in tumor cell density (blue in H&E staining) and apoptotic markers (brown in Cleaved Capase-3 and TUNEL) in Gem and NicNp combination treated PC tumors. *, ***, and **** indicate *p* < 0.05, 0.001, and 0.001, respectively, derived through One-way ANOVA. Histological images were captured at 10 × magnification

After confirming the synergy of Gem and NicNp in vitro, therapeutic efficacy of NicNp in combination with Gem in mice bearing tumors derived from AsPC-1 cells (athymic nude mice) and KCT-3248 cells (immunocompetent C57BL6 mice) was evaluated. The mice were treated weekly for 4 weeks with a combination of Gem and NicNp in separate injections given IP. The survival of KCT-3248 tumor-bearing mice treated with NicNp alone as well as in combination with Gem + NicNp was significantly improved at 22 days post-implantation of tumors with 95% surviving in the aforementioned groups while all the mice in the Np and Gem + Np groups died (Fig. 5a). Additionally, at the study endpoint which was 52 days after tumor implantation and 2 weeks after the last dose of Gem + NicNp, 25% of the mice in the Gem + NicNp group were still alive (Fig. 5a). The combination treatment of Gem + NicNp also reduced pancreatic tumor weights compared to Np and Gem + Np treated groups (Fig. 5b and S5, respectively). The KCT-3248 tumor tissue sections from Gem and Gem + NicNp treated mice were stained with H&E and cell death markers cleaved caspase-3 and TUNEL assay. Increased levels of cleaved caspase-3 indicate the induction of apoptosis, and was used as a marker in this study since Nic is known to increase apoptosis of cancer cells [18]. TUNEL is also a marker for apoptosis, specifically for excessive fragmentation of DNA [43]. Both cleaved caspase-3 and TUNEL were used to assess the efficacy of the combination treatment to kill cancer cells.

Staining with H&E showed reduced tumor cell density in the combination treatment group (Fig. 5c). Cleaved caspase-3 and TUNEL staining showed increased cell death in the Gem + NicNp treated mice (Fig. 5c).

## Discussion

Pancreatic cancer’s stagnant 5-year survival rate and poor clinical outcomes necessitate innovative treatments [1]. Current chemotherapy options for metastatic PC patients such as FOLFIRINOX, Gem, and Gem with nab-paclitaxel face challenges of systemic toxicity and drug resistance [6, 7]. To address this, we explored repurposing Nic, an FDA-approved anthelminthic drug known to target multiple oncogenic signaling pathways [16–18, 21]. However, free Nic has had limited applications in treating cancers partly due to its high degree of hydrophobicity, resulting in poor bioavailability [24]. This work evaluated novel biodegradable polyanhydride-based nanoformulations loaded with Nic (NicNp) to improve Nic biodistribution, improving its therapeutic efficacy in PC models in vitro and in vivo. Additionally, combination treatments of NicNp with Gem were examined and observed that the potential of Nic to sensitize PC cells to Gem synergistically.

Characterization of NicNp showed a moderate loading of Nic (12% wt), allowing for administering an effective dose within injection volume constraints for mice. NicNp exhibited a favorable Nic release profile for a once-weekly dose schedule, with the bulk of the Nic (~ 98%) released in two days (Fig. 1c). Our biodistribution experiments demonstrated that Nic injected IP in the form of NicNp is accumulated at relatively high concentrations of approximately 50 µg/g tissue and 10 µg/g tissue in pancreatic tumors derived from AsPC-1 and KCT-3248 cell lines, respectively, within 5 min post-injection and Nic was still detectable up to 72 h post-injection (Fig. 2a). Plasma concentrations of Nic in the same tumor-bearing mice were also detectable from 5 min to 72 h (Fig. 2b). The high tumor uptake of Nic from NicNp is likely due to leaky vasculature in tumors, known as the enhanced permeation and retention effect [44, 45]. Another reason for the high pancreatic concentration of Nic is that the NicNp was administered intraperitoneally. Colby et al., 2023, examined the intraperitoneal administration of drug-loaded nanoparticles for intraperitoneal mesothelioma cancer and showed a significant increase in the concentration of the nanoparticles in the intraperitoneal organs compared to the same nanoparticles administered intravenously [46]. In our study, higher Nic concentrations were measured in the spleen, liver, and pancreas from tumor-bearing mice treated with NicNp- compared to free Nic-treated mice (Fig. 2c). The opposite was true for Nic concentrations in non-intraperitoneal organs (kidneys, heart, brain, lungs) (Fig. 2c). Transport of NicNp out of the intraperitoneal cavity may be restricted due to their larger size while free Nic can more easily move out of the intraperitoneal cavity and into systemic circulation [47]. These results indicate that NicNp are more effectively accumulated in intraperitoneal organs compared to free Nic. Concerning the safety and toxicity of NicNp treatment given IP, there were no remarkable changes in the blood serum parameters of NicNp-treated mice within the first 24 h post-injection (Fig. S2b). There are no indications of systemic toxicity caused by NicNp treatment.

The efficacy of NicNp to increase survival and reduce tumor burden compared to free Nic was established in KCT-3248 tumor-bearing C57BL6 mice (Fig. 3). NicNp treatment increased survival, reduced tumor burden, reduced proliferation markers and induced apoptotic markers compared to both the negative control (Np) and free Nic treated mice (Fig. 3). There was no significant difference between free Nic and Np-treated mice in survival, tumor weight, or histopathology, which shows that free Nic was ineffective in treating PC without a nanocarrier (Fig. 3).

After establishing that NicNp is an effective nanocarrier, we examined NicNp’s ability to sensitize PC to Gem. Our studies found that NicNp, in combination with Gem, synergistically reduces tumor cell survival in vitro and in vivo. The synergy study confirmed again that NicNp is more effective in combination with Gem than free Nic (Fig. 4c). The most synergistic ratio tested in AsPC-1 cells was 1:2 Gem:NicNp with CI values all below 1 (Fig. 4c). This ratio is the most effective in reducing the Gem dose required to reach the IC50 and IC90 in AsPC-1 cells (Fig. 4a, b). The dose reduction index was favorable across all doses for both NicNp and Gem (Fig. 4d). Gem dose reduction is highly desirable because it minimizes off-target toxicity, prevents Gem resistance, and leads to cost savings [15]. About half as much Gem is required when combined with NicNp at a ratio of 1:2 to have the same cytotoxic effect in AsPC-1 cells as Gem alone (Fig. 4d). We confirmed the synergistic activity of NicNp with Gem in the orthotopic pancreatic tumor-bearing mice. The NicNp alone and combination treated NicNp + Gem group had significantly improved survival and decreased tumor burden with respect to the Gem + Np treated group (Fig. 5a). Additionally, the combination treatment group had significantly extended survival, with 80% of mice surviving 10 days after the last treatment and 25% surviving 21 days after the final treatment (Fig. 5a).

Several studies reported the encapsulation of Nic using nanoformulations for addressing various other cancers [24, 28, 29, 48, 49]. However, these formulations have not been studied in PC which is known to be more difficult to deliver drugs to than most other cancers [50]. Others have explored the potential of Nic to treat PC but very little has been done to address the practical limitation of its poor solubility [19, 51, 52]. Two studies have been published using the combination treatment of Nic and Gem, but neither of these studies used a nanocarrier for delivery of the Nic. The first study required DMSO to dissolve the Nic and administer the treatment twice a week IP [51]. The next study also dissolved the Nic in DMSO but used daily intragastric administration [19]. Our design is superior in that we were able to administer the NicNp once per week intra-peritoneally and an organic solvent like DMSO was not required. The current approach demonstrates stable niclosamide nanoparticles made using biodegradable polyanhydrides that showed optimal drug loading and release, and good efficacy in treating PC. Our study highlights the dose-sparing potential of using NicNp rather than free Nic in combination with Gem. Further, evaluations with targeted NicNp formulations can potentially enhance the delivery of Nic to pancreatic tumor cells, when delivered intravenously as opposed to IP. Though such evaluations complement our findings, current findings inevitably establish that NicNps are more effective than free Nic and sensitize pancreatic tumors to Gem synergistically. Clinical translation of NicNp will require scale-up of Nic-loaded polyanhydride nanoparticles (NicNp) synthesis by spray drying and following Good Manufacturing Processes. While CPH:SA nanoparticles have not been used in an FDA- approved drug formulation, the Gliadel® wafer implant, which is composed of the polyanhydride poly(carboxyphenoxy propane:sebacic acid) (CPP:SA) and the drug carmustine, has been FDA approved for the treatment of brain cancer.

## Conclusions

In summary, we have successfully synthesized biocompatible biodegradable polyanhydride nanoparticles loaded with the highly hydrophobic drug Nic for treating PC. Through comprehensive in vitro and in vivo experiments, these nanoparticles have proven to be an effective delivery system to increase the bioavailability and treatment efficacy of Nic. Toxicity studies in PC cell lines showed that NicNp reduced cancer cell survival significantly more than free Nic at the same dosage. In the biodistribution study, NicNp accumulated in the pancreas/tumor reaching peak concentration within 12 h post-treatment and remaining detectable for up to 72 h. Additionally, there were no obvious signs of systemic toxicity in serum parameters 24 h after NicNp treatment. In the in vivo efficacy studies, we established that NicNp is more effective in reducing tumor burden and increasing survival in mice compared to free Nic and, more importantly, that NicNp sensitizes pancreatic tumors to Gem treatment, resulting in a significant increase in survival of mice bearing orthotopic tumors. The combination of Gem with NicNp emerges as a promising therapeutic approach for PC, and further research is warranted to elucidate the underlying mechanism by which NicNp sensitizes PC to Gem.

## Supplementary Information

Below is the link to the electronic supplementary material.

Supplementary file1 (DOCX 402 KB)

## Data Availability

All data is available upon request by email to the corresponding authors.
