# Supplementary material for: Niclosamide-Loaded Polyanhydride Nanoparticles to Combat Gemcitabine Resistance in Pancreatic Cancer
Source: Regen Eng Transl Med. 2025 Mar 17;11(4):949–62. doi: 10.1007/s40883-025-00394-0 (PMC12852149; doi:10.1007/s40883-025-00394-0)
Supplement: Supplementary file 1 — Supplementary file1 (DOCX 402 KB) [file 40883_2025_394_MOESM1_ESM.docx]

**Regenerative Engineering and Translational Medicine**

**Supplementary Information**

**Title**: Niclosamide-loaded polyanhydride nanoparticles to combat gemcitabine resistance in pancreatic cancer

**Authors**

Brianna M. White^1^, Venugopal Gunda^2^, Susheel K. Nethi^1,7‡^, Nagabhishek Sirpu Natesh^3,4‡^, Adam S. Mullis^1^, Mariaelena Roman Sotelo^5^, Jeffrey North^5^, Chris Destache^6^, Balaji Narasimhan^1,7^, Surinder K. Batra^2^, Surya K. Mallapragada^1,7*^, Satyanarayana Rachagani^3,4*^

**Affiliations**

^1^ Department of Chemical and Biological Engineering, Iowa State University; Ames, Iowa, USA.

^2^ Department of Biochemistry and Molecular Biology, University of Nebraska Medical Center; Omaha, Nebraska, USA.

^3^ Department of Veterinary Medicine and Surgery, University of Missouri; Columbia, Missouri, USA

^4^ Roy Blunt NextGen Precision Health, University of Missouri, Columbia, Missouri, USA.

^5^ Department of Pharmacy Sciences, Creighton University; Omaha, Nebraska, USA

^6^ Department of Pharmacy Practice, Creighton University; Omaha, Nebraska, USA

^7^ Nanovaccine Institute, Iowa State University; Ames, Iowa, USA

*Corresponding authors. Email: suryakm@iastate.edu, srachagani@missouri.edu

‡ These authors contributed equally to this work

§ Current affiliation: Department of Biomedical Engineering, Tufts University, Medford, Massachusetts.


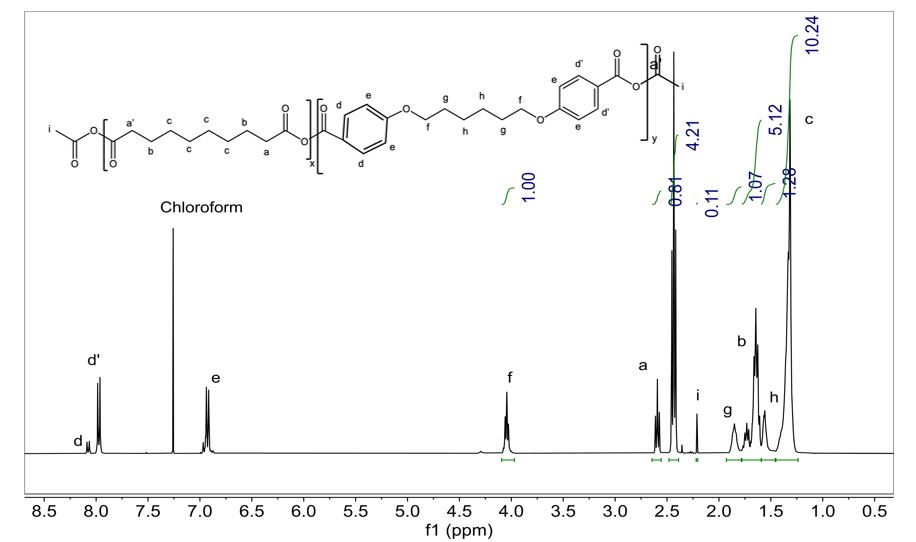


**Fig. S1** Integrated HNMR spectra of 20:80 CPH:SA labeled according to the chemical structure

**Fig. S2** Serological parameters measured from KCT-3248 tumor-bearing mice samples. Bar graphs represent mean ± SEM data derived from 3-10 biological replicates, * and ** represent p<0.05 and 0.01, respectively

**Fig. S3** Dose escalation study in orthotopic tumor-bearing mice. (a) Dose-dependent probability of survival of mice treated with the equivalent of 1.2, 1.8, and 3.0 mg Nic in NicNp formulation, (b) tumor weights of mice treated with escalating doses of NicNp, * represents p<0.05 from One-way ANOVA

**Fig. S4** Synergy studies in AsPC-1 cells in combination treatments of 1:1 and 1:2 Gem:NicNp. Combination index values for (a) 1:1 Gem:NicNp and (b) 2:1 Gem:NicNp determined using Compusyn software

**Fig. S5** Combination treatment of NicNp+Gem significantly reduced tumor weight in AsPC-1 tumor-bearing mice, *, **, and *** represents p<0.05, 0.01 and 0.001, respectively, from One-way ANOVA
